# JAKi Salvage Therapy Followed by Curative Cord Blood Transplantation in a XIAP-Deficient Infant with Relapsing HLH

**DOI:** 10.1007/s10875-023-01522-7

**Published:** 2023-05-20

**Authors:** Maria Elena Maccari, Camille Tron, Carsten Speckmann, the HSCT HLH study group, Julian Thalhammer, Stephan Ehl, Anke Peters, Brigitte Strahm

**Affiliations:** 1 Institute for Immunodeficiency, Center for Chronic Immunodeficiency, Medical Center-University of Freiburg, Faculty of Medicine, University of Freiburg, Freiburg, Germany; 2 Division of Pediatric Hematology and Oncology, Department of Pediatrics and Adolescent Medicine, Medical Center-University of Freiburg, Faculty of Medicine, University of Freiburg, Freiburg, Germany; 3 Univ Rennes, CHU Rennes, Inserm, EHESP, Irset (Institut de Recherche en Santé, Environnement et Travail) - UMR_S 1085, F-35000 Rennes, France; 4 Department of Biological Pharmacology, University Hospital of Rennes, Rennes, France; 5 Center for Pediatrics and Adolescent Medicine, Medical Center, University of Freiburg, Mathildenstr. 1, 79106 Freiburg, Germany

To the Editor:

X-linked inhibitor of apoptosis (XIAP) deficiency is a rare inborn error of immunity (IEI) caused by hemizygous mutations in *XIAP/BIRC4*. XIAP is important both in adaptive and innate immunity. Because of XIAP anti-apoptotic function in T cells, its deficiency is thought to limit antigen-specific T-cell expansion upon infection; moreover, XIAP is required for NOD2-signalling, crucial for intestinal immune homeostasis; finally, XIAP-deficiency results in dysregulation of NLRP3 inflammasome activation, leading to overproduction of pro-inflammatory cytokines [1].

In line with this complex pathophysiology, XIAP-deficiency is clinically characterized by various manifestations, ranging from localized inflammatory manifestations such as inflammatory bowel disease (IBD) to generalized, life-threatening hyperinflammation. Recurrent flares of hemophagocytic lymphohistiocytosis (HLH) are the most frequent clinical feature. A recent review [2] on 167 patients highlighted that outcome for conservatively treated patients is significantly worse for patients younger than 5 years at presentation (mortality > 40%).

Allogeneic hematopoietic stem cell transplantation (HSCT) currently offers the only curative treatment for XIAP-deficiency. Factors associated with an unfavourable outcome post-HSCT include myeloablative conditioning and poorly controlled HLH at transplantation [2]. Pre-HSCT treatment for HLH follows standard protocols. However, there is no consensus on optimal second-line treatment for the up to 25–50% of patients who fail to achieve remission with established regimens. The efficacy of Janus Kinase inhibitors (JAKi), i.e. ruxolitinib, in refractory HLH is increasingly reported and relies on the inhibition of signalling downstream of HLH-associated cytokines. However, the published reports focus on adult patients and we are not aware of previous reported experiences in pediatric-onset XIAP-deficiency [3].

We describe a one-year-old boy with a severe, early-onset XIAP-deficiency with recurrent, refractory HLH, successfully treated with ruxolitinib followed by HSCT at the age of 7.5 months using umbilical cord blood (UCB) from a mismatched unrelated donor (MMUD).

The boy is the first child of non-consanguineous Caucasian parents. Within the first three weeks of life, he developed failure to thrive, following acute-onset IBD. At the age of 2.5 months, he presented with afebrile pancytopenia (Hb 6.0 g/dl, platelets 13 G/L, leucocytes 2.69 G/L) and massive hepatosplenomegaly. Moreover, increased ferritin (4,201 ng/ml) and soluble interleukin-2 receptor (sIL2R; 6,612 U/ml) and decreased fibrinogen (121 mg/dl) were noticed, so that an HLH episode was suspected (5/8 criteria). The diagnostic work-up revealed deficient XIAP protein expression (Fig. 1a). Genetic analysis confirmed a previously reported [2] hemizygous mutation c.389_392del (p.Asp130Glyfs*11) in *XIAP* (Fig. 1b). Functionally, absence of TNFα production after L18-MDP-stimulation supported the pathogenicity of the mutation (Fig. 1c). An infectious trigger for the HLH episode was not identified. Interestingly, all four reported patients [2] with the same mutation also presented with HLH and three deceased either because of HLH or because of post-HSCT complications.

**Fig. 1:**
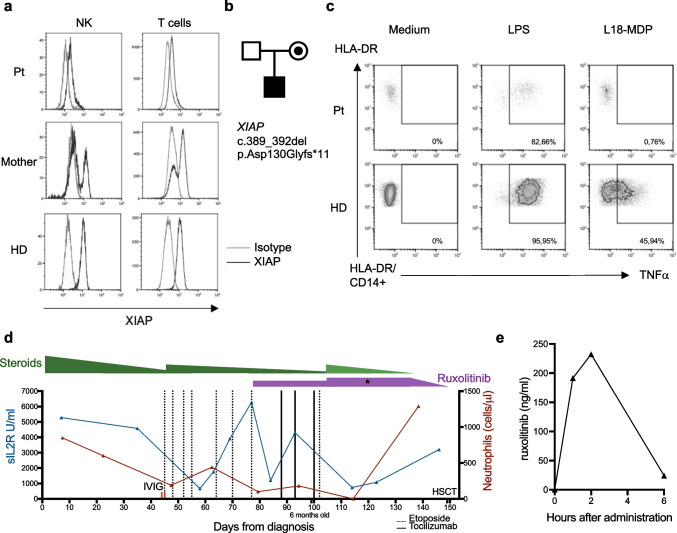
**a,** XIAP expression (black line) of patient (Pt), mother and healthy donor (HD) measured by flow cytometry on T cells and NK cells in whole blood. **b,** Family pedigree. **c,** TNFα production by monocytes (CD14 +) of patient (Pt) and healthy donor (HD) after stimulation with lipopolysaccharide (LPS) (positive control, 200 ng/ml) or L18-Muramyl dipeptide (MDP) (200 ng/ml), measured by flow cytometry. **d,** Overview of sIL2R and neutrophils under different medications. In dark green dexamethasone (10 mg/m^2^/d); in light green prednisolone (9 mg/m^2^/d). Etoposide (150 mg/m^2^/dose). Tocilizumab (12 mg/kg/dose). IVIG (1 g/kg/dose). * = timepoint of ruxolitinib PK-measurement. **e,** Ruxolitinib plasma-PK

Our patient had insufficient response to high-dose intravenous immunoglobulins (1 g/kg) and to first-line treatment (HLH-2004 protocol) with dexamethasone and etoposide (Fig. 1d). He experienced relapsing HLH activity following repeated tapering attempts of dexamethasone and after increasing the dosing intervals of etoposide due to severe neutropenia. We therefore initiated an “off-label” salvage therapy with tocilizumab (12 mg/kg/dose) and ruxolitinib (50 mg/m^2^/d) (Fig. 1d). Due to lack of dosing recommendations for this indication and age group, we followed the ruxolitinib dosing scheme for other indications, such as graft versus host disease (GvHD), and started treatment in 2 doses per day. Since treatment response was insufficient, dosing was increased to 75 mg/m^2^/d in 3 daily doses. Plasma pharmacokinetics (PK) assessment (method detailed in online resource 1) confirmed that dosing scheme change from 2 to 3 daily doses allowed reaching adequate between-dose exposure (Fig. 1e). A similar observation has been recently reported in a young patient with STAT3 gain-of-function disease [4]. The dosage adjustment resulted in prompt and sustained clinical response. Steroid treatment could be successfully weaned and complete control of HLH could be reached to proceed with HSCT at 7.5 months of age.

Yet, a matched family or unrelated donor could not be identified and the mother was excluded as haploidentical donor because of her mutation carrier status and skewed XIAP inactivation (Fig. 1a). The father and the extended family were also not eligible for stem cell donation. We extended the search to UCB and performed a salvage transplant procedure with a cryopreserved UCB graft from a 6/8 MMUD (9.64 × 10^5^ CD34 + cells/kg after thawing and washing). Prior to infusion, the patient received a reduced-intensity conditioning (RIC), following a recently published UCB protocol for IEI [5] with Fludarabin (5 × 1 mg/kg), Melphalan (2 × 70 mg/m^2^), Thiotepa (200 mg/m^2^), Alemtuzumab (2 × 0.5 mg/kg) and Hydroxyurea (9 × 30 mg/kg). Tacrolimus (0.075 mg/kg/d, weaned from day + 250) and mycophenolatmofetile (1200 mg/m^2^/d until day + 28) were given for GvHD prophylaxis. Ruxolitinib was weaned until day -1.

Nonetheless, the clinical course after HSCT was complicated by an acute-onset respiratory failure (day + 6) with non-infectious hyperinflammation (sIL2R 14,048 U/ml; Ferritin 100,680 ng/ml), which required invasive ventilation. He was treated with prednisolone (2 mg/kg/d), tocilizumab (4 × 12 mg/kg) and reintroduction of ruxolitinib (75 mg/m^2^/d in 3 daily doses). The reason for hyperinflammation remained unclear. Potential differential diagnoses include re-activation of XIAP-deficient tissue-macrophages, graft/engraftment-related responses following UCB HSCT, but also a ruxolitinib discontinuation syndrome. The early re-introduction of ruxolitinib did not interfere with hematological recovery after HSCT. The patient showed sustained engraftment with full donor chimerism and we obseverved a continous rise for all haematological lineages from day + 21 onward.

In our patient, inflammation stabilized after re-introduction of ruxolitinib, but at day + 16 and + 50 acute episodes of pulmonary haemorrhage occurred, which were treated with additional methyl-prednisolone pulses (25 mg/kg/d for 3 days). Both bleeding episodes occurred despite stable platelet counts > 40G/L. Following re-introduction of anti-inflammatory treatment, the patient recovered without prolonged sequelae and was discharged home on day + 88. Steroids were weaned until day + 80, ruxolitinib was reduced to 20 mg/m^2^/d but continued due to mild chronic skin GvHD. At last follow-up the patient was 2 years old, 18 months after HSCT and presented with mild erythema, covering approximately 30% of his body surface. His current treatment consists of topical prednicarbat and oral ruxolitinib (10 mg/m^2^/d at the last visit), which we gradually continue to taper. The patient shows normal development and is cured from the previous XIAP-associated symptoms.

In conclusion, our clinical observations suggest that JAK inhibition with ruxolitinib might be a promising and safe approach to control life-threatening peri-HSCT hyperinflammation in XIAP-deficient patients. The case also highlights that PK analyses may be helpful to optimize the individual dosing strategy, especially in young patients. Prospective randomized and disease-specific trials to further investigate benefits and risks of JAKi in hyperinflammatory IEI are urgently needed and should be combined with PK evaluations of ideal drug exposure at different ages.

## Supplementary Information

Below is the link to the electronic supplementary material.

Supplementary file1 (DOCX 13 KB)

## Data Availability

All data generated or analyzed during this study are included in this article.
