# Supplementary material for: JAKi Salvage Therapy Followed by Curative Cord Blood Transplantation in a XIAP-Deficient Infant with Relapsing HLH
Source: J Clin Immunol. 2023 May 20;43(6):1178–81. doi: 10.1007/s10875-023-01522-7 (PMC10354174; doi:10.1007/s10875-023-01522-7)
Supplement: Supplementary file 1 — Supplementary file1 (DOCX 13 KB) [file 10875_2023_1522_MOESM1_ESM.docx]

**Online Resource 1. Methodological summary ruxolitinib PK assessment**

Ruxolitinib exposure in plasma was assessed using a validated high performance liquid chromatography tandem mass spectrometry (LC-MS/MS) assay. Briefly, 100 µL of plasma were mixed with 300 µL of precipitant solution (acetonitrile-zinc sulphate in water (0.05 M), 1:1 v/v) and 100 µL of internal standard diluted in acetonitrile. Samples were vortexed for 1 min and centrifuged for 5 min at 2000g 4°C. A volume of 5µL of the supernatant was injected in a LC-MS/MS system (Ultimate 3000 and Quantis, ThermoFisher, [Waltham, Massachusetts, USA](https://www.google.fr/search?q=Waltham&si=AMnBZoFk_ppfOKgdccwTD_PVhdkg37dbl-p8zEtOPijkCaIHMjrOwoPM9hDMB6S9ndin1hg2iYOJftIs7F6s0tPrPWnJTgovlvP3udfl4XdpoUGNfP7s4P_Qj1B2zQFVRpZqKvzU5cNNjldpmAupMVuSmqmih4hA1dsAnmUwlykyQZBBWogIiH-sT_HrYhrkeBR0TUuRn5S4&sa=X&ved=2ahUKEwj_1qnMqer-AhUJTKQEHZwoCrcQmxMoAXoECFEQAw)). Ruxolitinib concentrations were measured at pre-dose, 1h, 2h and 4h post drug administration. The pharmacokinetics (PK) profile was obtained using results of the limited sampling strategy and population pharmacokinetics modeling adapted from Chen et al. (PMID: 23677817) in order to estimated ruxolitinib area under the curve of concentrations versus time between two administrations (AUC). The therapeutic range of ruxolitinib AUC in the context of HLH in XIAP deficiency is currently unknown, however, range of AUCs usually observed in pediatric patients in other indications can be found in reports from clinical studies (i.e. REACH studies in GvHD). This range helps the safe dose adjustment of ruxolitinib by identifying patient with deep under or overexposure compared to median exposure observed at usual dosing scheme. Our analysis focused primarily on an adequate between-dose exposure and was guided by clinical response.
